# Bioinformatics and Multiepitope DNA Immunization to Design Rational Snake Antivenom

**DOI:** 10.1371/journal.pmed.0030184

**Published:** 2006-06-06

**Authors:** Simon C Wagstaff, Gavin D Laing, R. David G Theakston, Christina Papaspyridis, Robert A Harrison

**Affiliations:** **1**Alistair Reid Venom Research Unit, Liverpool School of Tropical Medicine, Pembroke Place, Liverpool, United Kingdom; University of MelbourneAustralia

## Abstract

**Background:**

Snake venom is a potentially lethal and complex mixture of hundreds of functionally diverse proteins that are difficult to purify and hence difficult to characterize. These difficulties have inhibited the development of toxin-targeted therapy, and conventional antivenom is still generated from the sera of horses or sheep immunized with whole venom. Although life-saving, antivenoms contain an immunoglobulin pool of unknown antigen specificity and known redundancy, which necessitates the delivery of large volumes of heterologous immunoglobulin to the envenomed victim, thus increasing the risk of anaphylactoid and serum sickness adverse effects. Here we exploit recent molecular sequence analysis and DNA immunization tools to design more rational toxin-targeted antivenom.

**Methods and Findings:**

We developed a novel bioinformatic strategy that identified sequences encoding immunogenic and structurally significant epitopes from an expressed sequence tag database of a venom gland cDNA library of *Echis ocellatus,* the most medically important viper in Africa. Focusing upon snake venom metalloproteinases (SVMPs) that are responsible for the severe and frequently lethal hemorrhage in envenomed victims, we identified seven epitopes that we predicted would be represented in all isomers of this multimeric toxin and that we engineered into a single synthetic multiepitope DNA immunogen (epitope string). We compared the specificity and toxin-neutralizing efficacy of antiserum raised against the string to antisera raised against a single SVMP toxin (or domains) or antiserum raised by conventional (whole venom) immunization protocols. The SVMP string antiserum, as predicted in silico, contained antibody specificities to numerous SVMPs in E. ocellatus venom and venoms of several other African vipers. More significantly, the antiserum cross-specifically neutralized hemorrhage induced by E. ocellatus and *Cerastes cerastes cerastes* venoms.

**Conclusions:**

These data provide valuable sequence and structure/function information of viper venom hemorrhagins but, more importantly, a new opportunity to design toxin-specific antivenoms—the first major conceptual change in antivenom design after more than a century of production. Furthermore, this approach may be adapted to immunotherapy design in other cases where targets are numerous, diverse, and poorly characterized such as those generated by hypermutation or antigenic variation.

## Editors' Summary

**Background.:**

Of the 3,000 species of snakes worldwide, about 600 are poisonous; poisonous snakes are a particular problem in Africa and Southeast Asia. Because not all victims of snake bites get to hospital, estimates of illness and death caused are very approximate. However, one estimate quoted by the World Health Organization is that 2.5 million snake bites occur each year and 125,000 are fatal. The effects of snake bites vary, obviously depending on which snake does the biting, but immediate effects include swelling (around the bite or of other parts of the body), death of the area around the bite, and blood clotting problems. Nowadays, snake bite is treated with “antivenoms,” which are usually made from immunizing horses or sheep with snake venom. However, these antivenoms contain many different proteins that can themselves trigger unpleasant reactions in the recipient. One problem with developing antivenoms is that venoms contain many hundreds of different proteins, many of which may contribute to the toxic effect.

**Why Was This Study Done?:**

Recent scientific discoveries have led to new ways of finding which parts of an animal's genetic sequence are active in any one particular part of the body, and also whether the proteins produced from these genes are likely to cause illness. A snake's venom gland, where the venom is made, can be analysed this way. The researchers wanted to use this information to develop a more rational way of designing antivenoms.

**What Did the Researchers Do and Find?:**

They studied the venom glands of the carpet viper *(Echis ocellatus),* the most medically important snake in West Africa. They isolated expressed sequence tags (ESTs) produced by the venom glands. Each EST is a small part of the active part of a gene. They then focused on one group of genes that make proteins called snake venom metalloproteinases (SVMPs), which destroy other proteins, and which cause many of the severe symptoms, such as bleeding, seen after snake bite. They identified seven parts of these SVMPs that were likely to be clinically important, and engineered them into a single string of DNA. This product is known as an immunogen—that is, it can produce an immune response in an animal. And when this immunogen was injected into mice, the researchers found that the serum (the part of the blood that contains antibodies) from these mice did have a specific effect against the SVMPs in snake venom. It also had some effect, again in mice, against bleeding caused by small doses of snake venom.

**What Do These Findings Mean?:**

These results suggest that it is possible to use some of the newest genetic techniques to design immunogens that can be used to make highly specific, toxin-neutralizing antisera. These immunogens are a possible improvement over conventional antivenoms that are raised against whole venom. This approach could mean that lower doses of antivenoms would be needed than for conventional antivenoms. In addition, it may also be possible to design antivenoms that work against different species of snake venom. Results such as this may persuade a company that it is worth investing further in such antivenoms; as with many diseases of the poorer parts of the world, snake bites have not been of great interest to large pharmaceutical companies. In another paper published in *PLoS Medicine,* José María Gutiérrez et al. discuss the global problem of snake bites.

**Additional Information:**

Please access these Web sites via the online version of this summary at http://dx.doi.org/10.1371/journal.pmed.0030184

• World Health Organization page on animal bites, including snakes

• MedlinePlus Medical Encyclopedia pages of health information (these pages are most relevant in the US)

## Abstract 3

Seven epitopes were identified providing valuable sequence and structure/function information of viper venom hemorrhagins. This has created a new opportunity to design toxin-specific antivenoms.

## Introduction

Random sequencing of expressed genes (ESTs) often provides a rapid and affordable opportunity to gain a comprehensive insight into the biochemical complexities underlying many biological processes. Identifying the most clinically important toxins in venoms to inform the immunotherapeutic treatment of snake bite exemplifies this approach. Snake venom typically contains over 100 inter- and intraspecifically diverse proteins of varying toxicity that are difficult to purify in sufficient amounts for immunization and are often very poorly characterized at the protein sequence and functional levels. Whilst the increasing volume of genomic and transcriptomic data are undoubtedly beneficial here, our current ability to bioinformatically transform these data into novel therapies [ 1] is not well developed.

Conventional snake antivenom remains the only effective treatment of snake envenoming, but because of the above problems, current immunization protocols make no attempt to target the immune responses to the most clinically important toxins, but involve hyperimmunization of horses or sheep with whole venom. Variation in representation, immunogenicity, and toxicity of venom proteins means that (i) not all conventional antivenoms contain antibodies to important toxins [ 2], and (ii) dose-efficacy is reduced by antibodies to nontoxic components [ 3, 4]. This necessitates the administration of large volumes (30 to > 300 ml at 80 mg protein/ml) of antivenoms that increase the risk of early anaphylactoid and late serum sickness adverse reactions [ 5]. A systematic approach to select immunoprotective sequences for immunization using molecular sequence data alone would generate novel polyspecific antivenoms of high avidity, thus increasing dose efficacy and reducing toxicity to envenomed victims.

DNA immunization offers a rational approach to the design of toxin-specific immunotherapy, which we have previously demonstrated to induce titres and protective responses appropriate for antivenom production [ 6, 7] and generate antisera cross-reactive with venoms from phylogenetically distinct viper species and genera [ 8]. In light of the above and the sequence conservation of venom toxins, we proposed that a systematic approach to identifying common antigenic epitopes of venom toxins from venom gland EST databases would provide a rational approach to generating interspecific or intergeneric protective antibody responses satisfying the most desirable polyspecific properties of an antivenom.

As proof of principle, a comprehensive EST survey from the venom glands of *E. ocellatus,* the most medically important snake in West Africa, was undertaken and combined with a bioinformatic approach to identify epitopes of key structural or functional significance. We focused upon the group of snake venom metalloproteinases (SVMPs) responsible for the main, frequently lethal hemorrhagic effect of viper envenoming [ 9]. SVMPs are complex targets due to their multifunctional, multidomain nature and broad substrate specificity. SVMPs transcripts are classified by precursors encoding the metalloproteinase domain alone (class PI), metalloproteinase and C-terminal disintegrin domain (class PII), or disintegrin-like and cysteine-rich domains (class PIII) [ 10, 11]. Additional and extensive post-translational diversity of PII and PIII SVMPs also provide a reservoir in whole venom for a diverse array of processed disintegrins that interfere with normal mechanisms of tissue repair and hemostasis [ 12, 13]. Although their principal effect is the disruption of microvessel architecture by degradation of basement membrane components [ 14], this diversity endows SVMPs with a multiplicity of functions that are difficult to predict from transcriptional data alone. These functions include exacerbation of systemic bleeding by fibrinolytic activities, cleavage (and thus consumption) of coagulation factors, and disruption of platelet aggregation resulting in a variety of tissue-disruptive [ 14], coagulopathic, and haemostasis-disruptive mechanisms [ 15– 19] that contribute to the overall hemorrhagic pathology. Despite determination of the solution structures of several PI and PII SVMPs, the lack of structural information for PIII SVMPs leaves many questions concerning the structure/function relationships unanswered, particularly mechanisms of dimerization [ 20, 21] and their covalent addition to a C-type lectin domains generating the PIV isoforms [ 22, 23].

Here we describe for the first time (i) the design of a synthetic DNA immunization construct (EoSVMP string) containing a string of SVMP epitopes represented across numerous and diverse SVMP isoforms, (ii) the cross-generic and cross-specific antibody responses to multiple SVMPs induced by the EoSVMP string DNA immunogen, and (iii) the in vivo neutralization of venom-induced hemorrhage by the anti-EoSVMP string antibody.

## Methods

### Specimens and Venom

Venom glands were dissected from ten wild-caught adult specimens of E. ocellatus (Kaltungo, Nigeria) of random sizes (i.e., ages) and gender 3 d after venom extraction, when transcription is maximal [ 24]. Venom for immunoblotting was provided from pooled lyophilized venom extracted from wild-caught E. ocellatus (Kaltungo, Nigeria), C. cerastes (Egypt) *, Bitis arietans* (Ghana), and B. gabonica (Ghana) snakes maintained in the herpetarium of the Liverpool School of Tropical Medicine.

### cDNA Library Synthesis and Sequencing

Pooled venom glands were snap frozen then homogenized under liquid nitrogen, and total RNA was extracted using TriReagent (Sigma, Poole, United Kingdom). mRNA was purified using oligo-dt cellulose chromatography (Amersham, Buckinghamshire, United Kingdom). Ten micrograms of mRNA was used to construct a directional plasmid cDNA library of 1.4 × 10 ^6^ cfu/ml in Gateway CloneMiner (Invitrogen, Paisley, United Kingdom) using the first 480 ng of eluted cDNA from Sephacryl S-500 HR. Plasmid DNA isolated from 1,000 random colonies was sequenced (Lark Technologies, Essex, United Kingdom) using M13 forward primers. Full length sequencing of selected clones was achieved by single-stranded primer walking with custom forward and anchored oligo-dt reverse primers. These sequence data have been submitted to the EMBL database (see “Accession Numbers” below).

### Bioinformatics

Partial genome construction was undertaken on an Intel, Dual 2.8GHz RedHat Enterprise Linux 9.0 workstation running Biolinux 3.0 (available at: http://envgen.nox.ac.uk). Sequence chromatograms were processed using Trace2dbest (J. Parkinson, unpublished software) according to default parameters except polyA+ removal, which was set to 15. Clustering, assembly, and databasing of 1,000 sequences (100 sequences/round) was achieved by PartiGene [ 25] using CLOBB1 perl script [ 26] modified at source code to apply increased stringencies to initial pairwise comparisons of 100% over 50 bp. BLASTN/BLASTX annotation was achieved against databases of Uniprot, TREMBL, and all Serpentes sequences including ESTs and forward translations (minimum ORF of 200 nt) generated using GETORF within Jemboss [ 27]. Sequences were analyzed using CLUSTALX and Jameson-Wolf [ 28] (DNASTAR, Madison, Wisconsin, United States). In the absence of complete crystallographic data from class PIII SVMPs, surface exposure was confirmed by overlay using CD-search [ 29] on available models of class I Trimestatin [ 30] and the disintegrin Echistatin [ 31] (unpublished data). Peptide composition was refined using database 2 (db-2) (unpublished data).

### De Novo Gene Synthesis of Epitope Strings for DNA Immunization

Seven peptides were subjected to in silico concatenation (each peptide separated by two lysine spacers), reverse translation, murine codon optimization (available at: http://www.codon.jp), and fragmentation into thermodynamically balanced PCR primers for gene assembly using DNAWorks [ 32]. PCR gene assembly was performed according to previously described protocols [ 33] and recombination cloning into Gateway Multisite (Invitrogen) facilitated by the incorporation BP clonase recombination sites on the outermost assembly primers. Correct inserts, verified by sequencing, were subcloned upstream of an Igκ leader sequence in the DNA immunization plasmid pVaxSec, creating the construct hereafter referred to as EoSVMP string. The pVaxSec plasmid is a hybrid (unpublished data) we generated from a vaccine-appropriate kanamycin-selectable 3.0 Kb plasmid (pVax, Invitrogen) that lacked consensus Kozak, ATG start, and signal peptide domains. The latter were excised (NheI/HindIII) as a single sequence from an ampicillin-selectable, 5.2 Kb plasmid, pSecTag (Invitrogen) that we used in earlier studies [ 6].

### Preparation of Immunogens for GeneGun DNA and MAP Immunizations

To provide comparative immunological data to the sera raised by immunization with the EoSVMP-string DNA construct, mice were also immunized with DNA encoding the metalloproteinase (MP) domain ( Figure S1, 194–398), disintegrin (DC) domain (399–616), and metalloproteinase and disintegrin domains (MPDC) (194–616) of EOC00024 (EST: Eo_venom_05F04). Sequences were amplified by PCR with complementary 5′ and 3′ primers, and the amplicons cloned into pCR2.1 TOPO (Invitrogen) followed by subcloning into pVaxSec. Following sequence verification, transformed colonies were grown in 500 ml of LB medium, and plasmid DNA purified using MegaPrep plasmid DNA purification kit (Qiagen, Crawley, United Kingdom). Purified pVaxSec plasmid (vehicle control) and pVaxSec/MP, pVaxSec/DC, pVaxSec/MPDC, and pVaxSec/EoSVMP string were precipitated onto 1.6-μm gold beads and coated on the inner surface of half-inch lengths of plastic (Tefzel) tubing according to the manufacturer's protocol (Bio-Rad, Hercules, California, United States). The quantity of DNA gold beads was adjusted to provide individual tubing (shots) of 1 μg DNA/0.5 mg Au. The abdomens of anaesthetized female BALB/c mice (18–20 g) were shaved and each subjected to four “shots” expelled under a burst of helium gas at 350 psi into the epidermal layer using the Helios GeneGun (Bio-Rad). Groups of six mice were immunized with the four pVaxSec constructs or vector alone on weeks 0, 2, 4, 8, and 12 and sera examined after the final immunization. Multiple antigenic peptides (MAPs) for each epitope EoMPep 1–6 were synthesized by University of Birmingham (United Kingdom). Each MAP (100 μg resuspended in PBS), was homogenized in an equal volume of Freund's complete adjuvant (Sigma), and 25 μl injected into groups of female 18- to 20-g BALB/c mice ( *n* = 3) at four subcutaneous sites. Mice were boosted with MAPs without adjuvant on two subsequent occasions, 3 wk apart. Antisera against whole venom was raised in rabbits hyperimmunized with increasing doses of E. ocellatus whole venom initially in Freund's complete adjuvant over an established time period.

### Immunoblotting

Venom was separated by NUPAGE (Invitrogen) under reduced (8 μg, 4%–12% gradient NUPAGE, MOPS buffer) or nonreduced (10 μg, 12% NUPAGE, MES buffer) conditions. Following electrophoretic separation, gels were electroblotted onto 0.45 μm nitrocellulose (Bio-Rad) according to manufacturer's protocols. E. ocellatus venom (15 μg) in native sample buffer (62 mM Tris-Cl [pH 6.8], 30% glycerol, and 0.005% w/v bromophenol blue) was separated by Ornstein-Davis discontinuous native PAGE on 8% resolving gel (375 mM Tris-Cl [pH 8.8]) with a 4% stacking gel (125 mM Tris-Cl [pH 6.8]) at 200 V for 35 min in 250 mM Tris-Cl [pH 8.3] with 100 mM glycine followed by electroblotting in the same buffer to nitrocellulose at 100 V for 1 h using MiniProtean III (Bio-Rad). Following transfer, molecular mass markers were visualized by reversible staining with Ponceau S. Membranes were blocked in 20% soya milk in TBST (10 mM Tris-Cl [pH 8.5], 150 mM NaCl, and 0.1% Tween-20) overnight, followed by washes in TBST then incubation in test antisera at 1/200 dilution in 5% nonfat skimmed milk in TBST for 3 h at room temperature. Membranes were washed in TBST then incubated with a 1/1,000 dilution of HRP-conjugated goat anti-mouse immunoglobulins (Dako Cytomation, Cambridgeshire, United Kingdom) in TBST for 1 h at room temperature, then washed in TBST and developed using DAB peroxidase.

### ELISA

Maxisorp plates (96-well; NUNC, Denmark) were coated with whole E. ocellatus venom (100 ng/well) overnight at 4 °C in 0.05 M NaHCO _3_. Plates were washed with TBST and blocked for 1 h in 5% nonfat skimmed milk in TBST at room temperature. Individual murine sera were diluted at 1/200 then serially 1/5 in 5% nonfat skimmed milk, applied to wells, then incubated overnight at +4 °C. Plates were washed in TBST then incubated with a 1/1,000 dilution of HRP-conjugated goat anti-mouse IgG (Nordic, Tillburg, Netherlands) in TBST for 2 h at room temperature. Following washing in TBST, plates were developed using 0.02% 2,2′-azino-bis (Sigma) in phosphate-citrate buffer (pH 4.0) containing 0.015% H _2_O _2_, and absorbance measured at 405 nm using a Dynex OpsysMR plate reader.

### Neutralization of Venom Hemorrhagic Activity

The minimum hemorrhagic dose, defined as the amount of venom required to produce a 10-mm diameter hemorrhagic lesion 24 h after intradermal injection [ 34], was determined to be 10 and 8 μg/mouse for E. ocellatus and C. cerastes venom, respectively. These assays were performed according to WHO-approved protocols [ 4]. This amount of venom was preincubated with equal volumes of PBS (control) or test antisera for 30 min at 37 °C before intradermal injection into the dorsal skin of 18- to 20-g male CD-1 strain mice ( E. ocellatus venom: PBS, *n* = 3; rabbit anti- *E.* ocellatus venom, *n* = 3; DNA immunization constructs, *n* = 3; and C. cerastes venom: PBS, *n* = 3; rabbit anti- *E.* ocellatus venom, *n* = 3; pVaxSec, *n* = 4; EpiString, *n* = 3). Venom-induced hemorrhagic lesions that appeared on the inner surface of the skin 24 h later were coded and measured blind in two directions at right angles using digital calipers and background illumination. Percentage inhibition was calculated as the mean area zone of inhibition for experimental groups relative to vehicle controls (pVaxSec or PBS). A two-tailed Student's *t*-test was used to calculate *p*-values, assuming equal variance between two sample means. All animal experiments were performed using Home Office-approved protocols that have been reviewed and approved by the University of Liverpool local ethical review committee.

## Results

### The Bioinformatic Pipeline to Design Toxin-Neutralizing Immunotherapy

**Figure 1:**
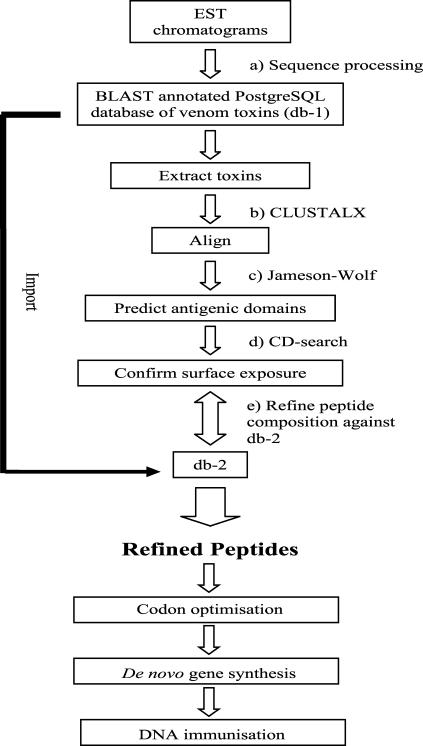
The Bioinformatic Pipeline Used to Identify Immunogenic Peptides EST chromatograms (A) are trimmed for contaminating sequences by trace2dbest, imported into PartiGene [ 25], clustered using CLOBB [ 26], BLAST-annotated, and finally used to populate db-1. Toxins are extracted and aligned using CLUSTALX (B) before (C) antigenic predictions by Jameson-Wolf (C) and three-dimensional superposition using CD-search (D). Peptide composition was refined against db-2 (E) before sequence incorporation into synthetic codon optimized DNA constructs for immunization (db, database).

The bioinformatic pipeline used for this project is described in Figure 1. Venom gland sequence chromatograms were processed and clustered using CLOBB1.pl [ 26] under conditions optimized to discriminate between venom gland EST isoforms. The first database, db-1, was populated by PartiGene [ 25] and contains a BLAST-annotated transcriptome for toxin cataloguing and identification. The composition of the entire annotated E. ocellatus transcriptome from db-1 is provided as Figure S2; toxins represented 47% of transcripts. Venom gland transcriptomes typically contain a high proportion of transcripts with no significant matches due to under-representation of snake venom toxins in public databases [ 35, 36] and will contain additional uncharacterized toxin components. Following extraction, antigenic profiling and alignment of selected toxins, a second, custom-designed data mining solution (db-2) scored epitopes for cross-reactivity against all ESTs and identified small antigenic peptides with broad predicted toxin-neutralizing potential. Peptides were concatenated in silico, separated by lysine spacers, and codon-optimized before de novo gene synthesis to create DNA immunogens.

### SVMPs Are Diverse and the Most Abundant Toxin Group in the E. ocellatus Venom Gland Transcriptome

**Figure 2:**
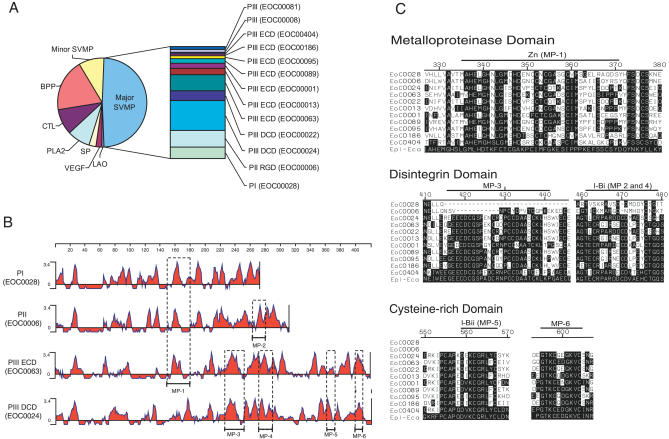
Identification of Conserved Domains in Snake Venom Metalloproteinases (A) Proportional representation of toxin-encoding transcripts. The proportional representation of SVMP clusters are expanded as a histogram and classified as PI–PIII isoforms. Identifiers for each cluster are indicated in parentheses. Minor SVMPs denote transcripts represented as singletons or smaller SVMP clusters (four ESTs or fewer) whose isoform class could not be reliably identified. BPP, bradykinin-potentiating peptides; CTL, C-type lectin; LAO, L-amino oxidases; PLA _2_, group II phospholipase A _2_; SP, serine proteinases; SVMP, snake venom metalloproteinases; VEGF, vascular endothelial growth factors. (B) Comparative Jameson-Wolf antigenic profiling of representatives of the PI (EOC00028), PII (EOC00006), PIII ECD motif (EOC00063), and PIII DCD motif (EOC00024). The propeptide domain, which is proteolytically released from the zymogen [ 9], is excluded. Antigenic domains that show the greatest levels of sequence conservation are indicated below each transcript (MP 1–6). (C) Sequence alignment of E. ocellatus venom gland SVMP isoforms. The alignments are truncated and separated into three SVMP functional domains annotated with antigenic domains (EoMP 1–6) and motifs of functional or catalytic significance. Residues identical to ecarin (Epl–Eca [Q90495]) are shown in black for comparison. Full-length alignments are provided in Figure S1. I-Bi, integrin-targeting tripeptide motifs; I-Bii α _2_β _1_ integrin-binding VKC-like motifs in jararhagin-C [ 46]; Zn, catalytic zinc-binding motif.

In order to obtain a global panorama of the toxin composition of E. ocellatus venom, ESTs were separated into toxin families according to their BLAST similarity against Uniprot/TREMBL databases. SVMPs dominated the toxin-encoding profile (58%, 243 transcripts) relative to other toxin groups ( Figure 2A), encoding isoforms from all the three (PI-PIII) major SVMP isoform classes [ 10, 11]. The relative expression levels indicate that this transcriptome is dominated by PIII isoforms relative to PI- and PII-encoding transcripts ( Figure 2A). These clusters represent 88% of all expressed SVMPs, the remainder present as single copies. Because clustering of venom toxin isoforms is especially vulnerable to chimeric assemblies, two random clones from each pentapeptide SVMP cluster were sequenced. This confirmed accurate assembly and resulted in the characterization of 11 novel SVMP transcripts collectively accounting for 72% of SVMPs. The number and diversity of PIII isoforms, the most potent hemorrhagins [ 9], is notable and consistent with the hemorrhagic pathology of victims bitten by this viper [ 37, 38]. Full-length comparative sequence analysis of the PI–PIII EoSVMP isoforms (provided in Figure S1) illustrates the high degree of sequence and domain conservation that characterizes the viper venom hemorrhagins [ 10, 39]. The molecular diversification of the E. ocellatus SVMPs far exceeds that reported in the venom glands of other viper species [ 35, 36, 40]. Our cDNA library was intentionally constructed from a pool of venom glands without normalization to provide transcriptomic data that best reflects venom composition, including important isoform-distinct sequence differences and toxin expression bias. It is therefore not clear whether this diversity is consistently reflected in individuals or results from population variation in venom composition reflecting differences in age (size), diet, and availability of local prey [ 41, 42].

### EoSVMP String Construction

The diversification described above indicated that neutralization of the hemorrhagic activity of whole venom will require antibodies targeting surface-available and antigenic epitopes contained within most, if not all, the numerous and diverse SVMP isoforms. Since only limited structural information is available for SVMPs, we first performed Jameson-Wolf antigenic profiling of representatives from each isoform class (PI–PIII). This analysis identified six domains (EoMP 1–6) whose antigenic profiles are similarly conserved across isoforms ( Figure 2B). These antigenic domains aligned to functionally significant domains ( Figure 2C) as follows: EoMP 1, the catalytic zinc-binding motif (identified as Zn in Figure 2C); EoMP 2, integrin-targeting tripeptide motifs containing α _5_β _1_ RGD (I-Bi) [ 43]; EoMP 4, containing an α _2_β _1_ ECD motif [ 44, 45] substituted for DCD motifs in EOC00024 and 22 (also I-Bi); and EoMP 5, which contains α _2_β _1_ integrin binding VKC-like motifs in jararhagin-C (IBii) [ 46]. Two additional domains of unascribed functions, EoMP 3, the GEECDC box upstream of IB-i, and EoMP 6, a C-terminal region in the cysteine-rich domain, were also selected on the basis of their unusually high level of interspecific and intergeneric sequence conservation [ 8, 39] and high potential immunogenicity.

**Figure 3:**
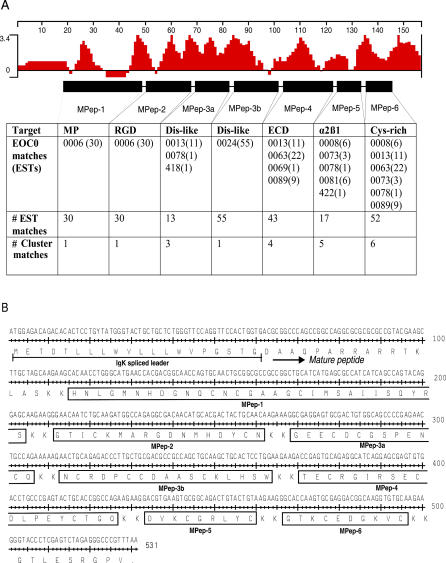
The EoSVMP String (A) Upper graphic: Jameson-Wolf antigenic profiling of the mature EoSVMP string synthetic DNA immunization construct. Lower chart: Target domains for individual MP epitopes. Cluster matches against each peptide are shown. The number of ESTs in each cluster is indicated in parentheses. α2β1, α _2_β _1_ integrin-binding motif in Jararhagin-C; Cys-rich, C-terminal region of the cysteine-rich domain; Dis-like, disintegrin-like domain; ECD, ECD motif and flanks; MP, metalloproteinase catalytic domain; RGD, RGD-containing PII and flanking sequences. (B) Complete nucleotide and coding sequence of EoSVMP string. Sequence and locations of epitopes, separated by lysine spacers are indicated.

Multiple sequence alignment of the antigenic domains EoMP 1–6 from all the full-length SVMP isoforms characterized from this cDNA library demonstrated extensive sequence diversification of these domains between isoforms ( Figure 2C). This suggested that immunization with antigenic domains (EoMP 1–6) from a small number of SVMPs would be unlikely to induce potent cross-reactive antibody responses capable of neutralizing venom-induced hemorrhage. To create an antiserum that would react with epitopes representative of all the SVMP isoforms, an additional data-mining solution, database db-2, was developed to select a single epitope from each antigenic domain present in the maximum number of SVMP isoforms. Db-2 was used to evaluate the frequency of peptides in the E. ocellatus venom gland EST database (EoVgDbEST) by taking EST expression bias (numerical EST representation and cluster diversity) into account by reporting (i) the number (and size) of clusters matched to each candidate query peptide, and (ii) the total number of individual ESTs matched to each peptide. Using db-2, the length of each antigenic domain (EoMP 1–6) was incrementally reduced and the peptide composition refined into seven peptides (EoMPep 1–6) that represented the maximum number of matches to SVMP clusters and ESTs ( Figures 2A and 3A). It should be noted that because of the sequence divergence within the centre of domain EoMP 3 ( Figure 2C, residues 415–440), this domain was separated into two overlapping peptides, EoMPep 3a and 3b, which together scored higher, by db-2, than a single peptide spanning domain EoMP 3.

EoMPep 1–6 were synthesized, by de novo gene synthesis, as a murine codon-optimized, single, multi-epitope sequence (EoSVMP string) that was then inserted into the DNA immunization plasmid pVaxSec. Two lysines were inserted between each EoMPep to serve (i) as spacers and (ii) as sites for intracellular proteolytic cleavage by the DNA-transfected epidermal cell, thus promoting their antigen presentation as single epitopes. Figure 3B shows the primary sequence, high antigenic profile and location of epitopes in the EoSVMP string.

### EoSVMP String Induces Antibody Responses to Multiple SVMP Isoforms

**Figure 4:**
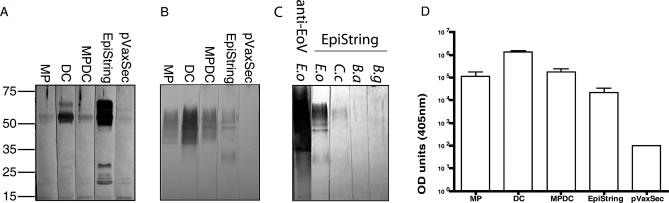
EoSVMP String Antiserum Recognizes Multiple SVMP Isoforms (A and B) Comparative reactivity on immunoblots using pooled murine antisera raised against EOC00024 domains (MP, DC, and MPDC) and EoSVMP string to E. ocellatus venom under reduced SDS-PAGE (A) and native PAGE (B) conditions. (C) Comparative reactivity on native PAGE immunoblots of EoSVMP string antiserum to venoms from E. ocellatus (Eo), C. cerastes (Cc), B. arietans (Ba), and B. gabonica (Bg). Cross-reactivity of rabbit antisera against E. ocellatus venom (anti-EoV) is shown for comparison. (D) IgG ELISA end-point titres determined by serial dilution of murine antisera. Values represent the mean dilution (± standard error of the mean) of sera equaling the titre of sera from unimmunized BALB/c mice.

To provide comparison between (i) this SVMP epitope-string immunization and (ii) our earlier single SVMP-immunization approaches to generate toxin specific and venom neutralizing antibody [ 6], we also immunized mice with pVaxSec constructs expressing the metalloproteinase domain only (MP), the disintegrin domain only (DC), or metalloproteinase and disintegrin domains (MPDC) of the SVMP transcript, EOC00024. This EST contig was selected as a PIII SVMP paradigm for this comparison due to its highest representation (55 ESTs) over other clusters ( Figure 2A) and its similarity with other PIII SVMPs in EoVgDbEST, and would therefore be expected to provide immunological cross-reactivity to many SVMP isoforms. Sera from mice immunized with the constructs expressing the MP, DC, or MPDC domains of EOC00024 ( Figure 4), however, showed restricted specificity to isoforms mainly in the 55- to 60-kDa molecular mass range ( Figure 4A) but high titres against whole venom as determined by ELISA ( Figure 4D). In comparison, and according to bioinformatic predictions, sera from mice immunized with the EoSVMP string showed intense and, importantly, extensive immunoreactivity to diverse SVMPs in wide molecular mass ranges of 50–70 kDa and 20–30 kDa ( Figure 4A). EoSVMP string antiserum, however, showed a lower ELISA titre and less intense native immunoblot reactivity to whole venom than EOC00024 antisera ( Figure 4B and 4D) or rabbit anti- E. ocellatus venom ( Figure 4C), and it was only weakly reactive against the venom of C. cerastes ( Figure 4C). Whilst the intensity of immunoreactivity is higher against reduced venom proteins than native proteins, the multiplicity of venom components reactive with the EoSVMP string antiserum was similar ( Figure 4A– 4C). This suggests that the EoSVMP string induces polyspecific antibody responses capable of binding to both linear and native, conformationally intact SVMP epitopes.

### EoSVMP String Induces Cross-Generic and Cross-Specific Antibody Responses

**Figure 5:**
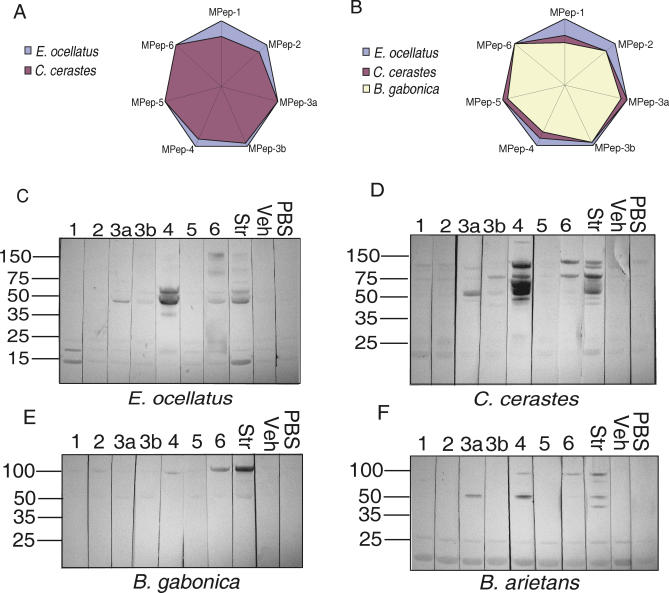
Prediction and Experimental Verification of the Immunological Conservation of EoMPep 1–6 in Phylogenetically Distinct African Viper Species (A and B) The immunological conservation of EoMPep 1–6 is predicted using radar charts comparing the percentage highest bit score results generated by BLAST searches of EoMPep 1–6 against the E. ocellatus venom gland EST database (VgDbEST) (taken as 100%) with BLAST searches against VgDbEST databases from (A) C. cerastes (SCW, unpublished data, 2006) and (B) the latter and B. gabonica [ 35]. (C–F) Experimental assessment of immunological cross-reactivity using pooled sera raised against EoMPep 1–6 as individual MAPs (indicated as numerals 1–6 above each blot) and EoSVMP string (Str) following nonreduced immunoblotting of whole venom of four viper species (indicated below each blot). Molecular mass markers are shown to the left. Veh, pVaxSec DNA vehicle control.

We interrogated other viper venom gland EST databases to predict the extent to which epitopes (EoMPep 1–6) are conserved in venoms of other medically important African vipers. This analysis, with the exception of EoMPep 1 and 2, predicted conservation of the majority of epitopes in C. cerastes ( Figure 5A), but only EoMPep 6 in the gaboon viper, Bitis gabonica ( Figure 5B). Sera from mice immunized with individual MAPs of EoMPep 1–6 were used to confirm these predictions. All epitopes, except EoMPep 2 and 5, were detected with these antisera in the venom of phylogenetically distinct vipers distributed throughout Africa: E. ocellatus (West Africa) ( Figure 5C), C. cerastes (North Africa) ( Figure 5D), B. gabonica ( Figure 5E), and the puff adder, B. arietans (pan-African distribution) ( Figure 5F). As predicted, EoMPep 1 was restricted to *E. ocellatus,* whereas EoMPep 3a and EoMPep 3b were immunologically conserved between E. ocellatus and *C. cerastes;* the latter sera also reacted with components of B. arietans venom, but not with those in the venom of the related species, B. gabonica. There were notable differences in intensities; EoMPep 4 antiserum was intensely reactive with venom components from E. ocellatus and C. cerastes but not with B. gabonica and *B. arietans,* whereas the converse was true for EoMPep 6. Most significantly, the EoSVMP string antiserum consistently recognized more SVMP isoforms in all venoms than antisera raised against single epitopes. Taken together, these results indicate that the EoSVMP string DNA immunogen induces antibody responses to multiple conserved epitopes, but with intensities that vary with expression levels or antigenicity of its constituent epitopes in venoms of different vipers.

### Sequence-Structure Relationships between Viper Venom Hemorrhagins

The EoMpep 4 (SECD motif) is highly conserved in PIII hemorrhagins, whose extensive level of sequence homology and domain structure would be expected to produce comparable mobilities on Western blots. Antisera to EoMPep 4 and EoMPep 6 (C-terminal motif), however, both showed cross-reactivity to distinct, and larger SVMP molecular mass variants that are likely to be multimeric SVMPs ( Figure 5C– 5F). While the function of the EoMPep 3a, 3b, and 6 domains remains unresolved, their extraordinary cross-generic conservation suggests biological significance. Structurally, the few multimeric SVMPs reported to date—the homodimeric PIII SVMPs VAP1 and HV1 [ 20, 21] and the heterodimeric PIII (PIV) with an additional disulphide C-type lectin domain [ 22, 23, 47]—all contain an additional cysteine residue. As a result, it has been implied that the additional cysteine residue facilitates multimerization [ 10, 11]. With the exception of EOC0001, however, which contains an additional Cys-367 ( Figure S1) in common with VAP1 and HV1 [ 20, 21] and consequently may exist as a native dimer, the majority of ECD motif-containing PIII SVMPs contain a cysteine ratio of 6:16:12 (metalloproteinase, disintegrin-like, and cysteine-rich domain) ( Figure S1) consistent with monomeric, nonprocessed SVMPs [ 10, 13]. These data suggest that an additional cysteine residue is not necessarily required for assembly of multimeric SVMPs that may occur via isomerization of existing disulphides.

### EoSVMP String Antiserum Substantially Inhibits Venom-Induced Hemorrhage In Vivo

**Figure 6:**
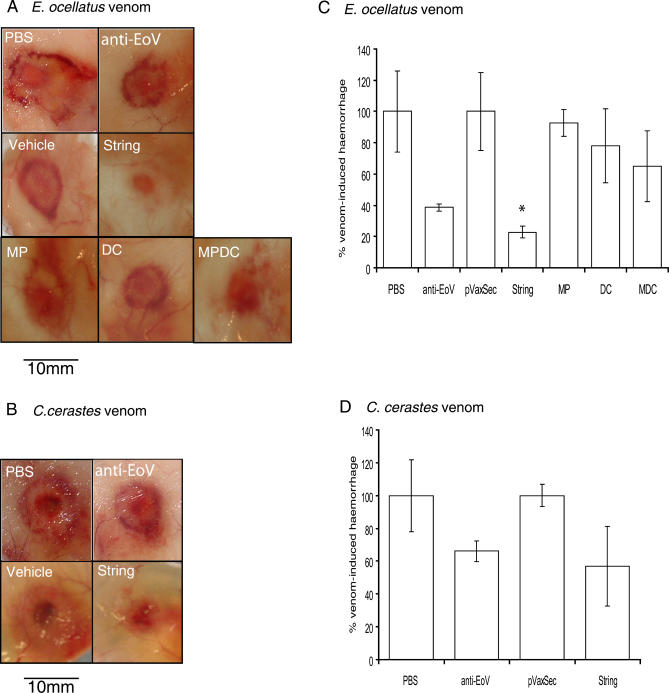
Neutralization of Venom-Induced Hemorrhage (A and B) Representative hemorrhagic lesions in the skin of mice injected with one minimum hemorrhagic dose of E. ocellatus venom (A) and C. cerastes venom (B) following preincubation with PBS, rabbit anti- E. ocellatus venom (anti-EoV), or pooled sera from mice immunized with the indicated DNA immunogens. (C and D) The above data expressed as mean standardized area of hemorrhage on the inner surface of the skin (± 1 standard error of the mean). Values are expressed mean percentage hemorrhage relative to control, pVaxSec (DNA immunogens), or PBS (protein immunogens) which was taken as 100%. A result statistically significant relative to controls is denoted by an asterisk.

The comparative venom hemorrhage-neutralizing efficacy of antibodies raised by DNA immunization was assessed using a preclinical minimum hemorrhagic dose assay [ 4], measured by a blinded operator. These antibodies were compared to sera raised by conventional (whole venom) immunization protocols using rabbit anti- E. ocellatus venom. Antisera against the EOC00024 MP domain was ineffective at neutralizing E. ocellatus venom-induced hemorrhage ( Figure 6A), and EOC00024 DC and MPDC antisera apparently offered limited, but statistically nonsignificant, protection against E. ocellatus venom-induced hemorrhage. In comparison, the EoSVMP string antiserum resulted in a 75% ( *p* = 0.04) reduction in the mean area of hemorrhage induced by E. ocellatus venom ( Figure 6A and 6C) relative to the DNA immunization control, pVaxSec. This reduction compared favorably with that effected by the rabbit anti- E. ocellatus venom (60%) ( *p* = 0.08), relative to PBS control.

Bioinformatic analysis ( Figure 5A) implying that the SVMP string antiserum would also neutralize the hemorrhagic activity of C. cerastes venom was experimentally confirmed. Thus, the EoSVMP string antiserum neutralized the in vivo hemorrhagic activity of C. cerastes venom by 43% ( Figure 6B and 6D) compared to rabbit anti- E. ocellatus whole venom (34%) relative to immunization vehicle controls, although below the level of statistical significance.

An almost complete reduction in the intensity, rather than just the size, of the hemorrhagic lesions induced by EoSVMP string antiserum is also noteworthy ( Figure 6A and 6B), because this is not reflected in the statistical comparisons of lesion size ( Figure 6C and 6D). Reduction in the intensity of lesions with pVaxSec (DNA control) relative to positive control (PBS preincubated) venom was expected and reflects baseline inhibition of some PIII SVMPs by endogenous natural substances in mouse sera such as α _2_-macroglobulin [ 48]; this is unrelated to specific immunoglobulin activity.

## Discussion

Using a bioinformatic approach, we constructed a multiepitope DNA immunogen that generated antibody responses that neutralized venom hemorrhagic toxins, despite the extensive intraspecific diversity of viper venom hemorrhagins. This epitope-directed approach to the design of antivenoms is a considerable advance over current (whole venom) immunization protocols and is a logical progression from our previous studies using the disintegrin-like domain of a single SVMP as an immunogen [ 6].

The precise mechanisms of venom-induced hemorrhage are not completely understood. Individually characterized PIII SVMPs from different species have been shown to be responsible for many of the diverse tissue-disruptive, coagulopathic, and haemostasis-disruptive mechanisms that contribute to hemorrhagic pathology (extensively reviewed recently in [ 12, 14, 16– 18]). Here we provide evidence implicating a multiplicity of SVMPs in the hemorrhagic pathology of E. ocellatus envenoming that exceeds estimates of other viper species reported to date [ 35, 36, 40]. The diverse functions effected by E. ocellatus SVMPs is illustrated by the sequence similarity (including disulphide-bonded architecture) of (i) the PIII EOC00404 to the prothrombin activator ecarin [ 49, 50], and of (ii) the PII SVMP EOC00006 to the fibrinolytic metalloproteinase lebetase [ 10, 51], purified from venom that lacks a disintegrin domain [ 52]. Furthermore, the disintegrin domain of EOC00006 is also identical to the platelet aggregation-inhibiting disintegrin ocellatusin [ 43]. This suggests that the mature EOC00006 is a proteolytically cleaved precursor of ocellatusin and a metalloproteinase with fibrinolytic activities. Most notable is the abundance and diversity of PIII SVMPs containing the ECD motif. This motif in jararhagin (an SVMP of Bothrops jararaca venom) has been shown to inhibit collagen-induced platelet aggregation via α _2_β _1_ [ 45]. The SECD motif has also been shown to be important in ADAM-1 and 2-mediated sperm-egg interactions [ 53, 54]. Further variations are introduced in the homologous PIII transcripts, EOC00024 and EOC00022, in which the SECD domain is substituted for DDCD. Whilst the ligand specificity of each of these peptides requires experimental verification, taken together these data indicate that multiple mechanisms are synergistically deployed to achieve hemorrhagic and systemic bleeding, and therefore a comprehensive strategy of toxin epitope-targeted inhibition is required to develop an effective antivenom.

In light of this diversification, generating toxin-neutralising antisera by using strings of epitopes has not previously been attempted. This, in part, is due to the assumption that monospecific immunotherapeutic antibody responses are unlikely to be clinically effective against such diverse venom toxins, and partly because a system to identify epitopes in large databases has not previously been developed. The development of robust algorithms to predict the function of individual, uncharacterized toxin transcripts (especially PIII SVMPs) has indeed proved difficult [ 55, 56], and because of the multimeric nature of many venom toxins we approached the problem of designing toxin-neutralizing immunotherapy by developing a multi-isoform transcriptomic (i.e., based on ESTs) approach. We designed and evaluated a systematic approach to select immunoprotective sequences that provide the maximal immunological cross-reactivity and toxin-neutralizing efficacy to the majority of isoforms in a multi-isoform dataset. Importantly, the approach described here circumvents the absolute requirement for prior structural and functional information compared to the majority of existing approaches [ 57], which typically search for T or B cell epitopes [ 58] or preexisting motifs (such as those from secretory or membrane associated proteins) [ 59– 61], or which require other prior experimental evidence such as data derived from expression library immunization screening protocols [ 62]. The novelty of this epitope selection approach lies in the direct use of multiepitope expression bias inherent in non-normalised EST databases to score epitopes and inform the design of DNA immunogens that are “epitopically balanced” by library representation. This is achieved principally by constraining the consensus residues of antigenic domains (for example MP-4 TXCRXXRXECDL) to provide a scaffold upon which degenerate positions (X) are mutated in silico until the highest-scoring immunogenic peptide is identified. By incorporating an analogous frequency matrix that considers antigenic mutation/variation within a geographical region, this principle could also be applied to refining the peptide composition of subunit vaccine candidates to produce those with broader geographical specificity.

Antiserum raised against the engineered EoSVMP string DNA immunogen, in accordance with bioinformatic predictions, substantially neutralized hemorrhage induced by the venoms of two vipers, E. ocellatus and *C. cerastes,* which have markedly distinct distributions (North and West Africa) and habitats [ 37, 63]. The venom neutralization achieved by the EoSVMP string antiserum compared favorably with that of antiserum raised in rabbits by conventional (whole venom) immunization protocols, despite its lower titre and intensity on native Western blots. This indicates that the titre of a toxin-specific antibody is not necessarily a measure of its toxin-neutralising efficacy. Whilst lower titres presumably reflect the incorporation of a smaller number of epitopes into the immunogen, the toxin-neutralising efficacy of the EoSVMP string antiserum suggests that these selected epitopes are (i) of functional significance and (ii) generate antibody to native venom toxins.

The cross-generic neutralization observed in this study may result from the broad modality of functional inhibition designed into the DNA immunogen, rather than from mechanistic similarities of hemorrhage between these two vipers *.* Comparative Western blots between the EoSVMP string and antisera raised against individual epitopes as MAPs demonstrate that the diverse antigen reactivity profile of EoSVMP string antisera is contributed by EoMPep 1, 3a, 3b, 4, and 6, and that EoSVMP string also recognises EoMPep 2 and EoMPep 5 MAPs on dot immunoblots (unpublished data). The immunoreactivity of EoSVMP string antisera therefore contains contributions from antibodies specific to all seven epitopes, indicating the correct processing and antigen presentation of each constituent epitope of the EoSVMP string. In comparison, antibodies to the DC domain of EOC00024 did not markedly neutralize E. ocellatus venom-induced hemorrhage, despite being of higher titre and the success of a similar approach using the DC domain of jararhagin, that neutralized over 70% of B. jararaca venom-induced hemorrhagic activity [ 6]. These data indicate that inhibition of venom-induced hemorrhage is not simple and that many antibodies are not clinically beneficial because, despite being of high titre, they fail to neutralize the functional activity of critically important SVMP domains.

Our successful use of bioinformatics analysis in selecting epitopes to create an epitope-string DNA immunogen that generate antibodies capable of neutralizing the function of a multi-isomeric group of venom toxins is, to our knowledge, the first demonstration that a single synthetic immunogen can replicate the toxin-neutralizing capabilities of conventional antivenom. We believe that this new rational basis to the design of immunotherapy for snake bite can be expanded to provide a polyspecific antivenom for disparate venomous snakes localized within a defined geographical region. We are, therefore, currently creating epitope-string DNA immunogens to generate antibodies designed to neutralize the function of SVMPs, phospholipases A _2_ [ 64, 65], serine proteases [ 19], C-type lectins [ 66, 67], and disintegrins present in the venom gland transcriptomes of E. ocellatus [ 68] *, B. arietans,* and *C. c. cerastes,* the vipers responsible for most deaths by snake bite in Africa. We believe that such an antivenom, because of its exquisite toxin specificity, will have improved toxin-neutralizing IgG titres and therefore pose less risk of anaphylactoid and serum sickness adverse effects than conventional antivenom.

## Supporting Information

Figure S1 Comparative Sequence Analysis of Full-Length PI-PIII SVMP Isoforms (144 KB PDF)

Figure S2 Composition of the E. ocellatus Venom Gland Transcriptome
(83 KB DOC)

### Accession Numbers

The sequences described here have been submitted to EMBL under the following accession numbers: EOC00028 (AMO39698), EOC00006 (AMO39693), EOC00024 (AMO39697), EOC00063 (AMO39692), EOC00022 (AMO39696), EOC00013 (AMO39695), EOC00001 (AMO39691), EOC00089 (AMO39699), EOC00095 (AMO39694), EOC00186 (AMO39700), and EOC00404 (AMO39701). The structure of Trimestatin was obtained from PDB accession number 1J2L.

## Abbreviations

db: database
DC: disintegrin
EoVgDbEST: E. ocellatus venom gland EST database
EST: expressed sequence tag
MAP: multiple antigenic peptide
MP: metalloproteinase
SVMP: snake venom metalloproteinase
VgDbEST: venom gland EST database
