# Correction: Bioinformatics and Multiepitope DNA Immunization to Design Rational Snake Antivenom

**DOI:** 10.1371/journal.pmed.0050209

**Published:** 2008-10-28

**Authors:** Simon C Wagstaff, Gavin D Laing, R. David G Theakston, Christina Papaspyridis, Robert A Harrison

Correction for:

Wagstaff SC, Laing GD, Theakston RDG, Papaspyridis C, Harrison RA (2006) Bioinformatics and multiepitope DNA immunization to design rational snake antivenom. PLoS Med 3(6): e184. doi:10.1371/journal.pmed.0030184

In the Supporting Information section of this paper, Figure S1 and Figure S2 were incorrectly linked. Figure S1 and Figure S2 labeled and in the order originally intended are provided here.

## 

Figure S1 Comparative Sequence Analysis of Full-Length PI-PIII SVMP Isoforms (48 KB PDF).

Figure S2 Composition of the E. ocellatus Venom Gland Transcriptome Found at doi:10.1371/journal.pmed.0050209.sg002 (32 KB PDF).

