# Supplementary material for: Bioinformatics and Multiepitope DNA Immunization to Design Rational Snake Antivenom
Source: PLoS Med. 2006 Jun 6;3(6):e184. doi: 10.1371/journal.pmed.0030184 (PMC1472699; doi:10.1371/journal.pmed.0030184)
Supplement: Supplementary file 1 [file pmed.0030184.sg001.pdf]

Figure S2: Composition of the *E.ocellatus* venom gland transcriptome

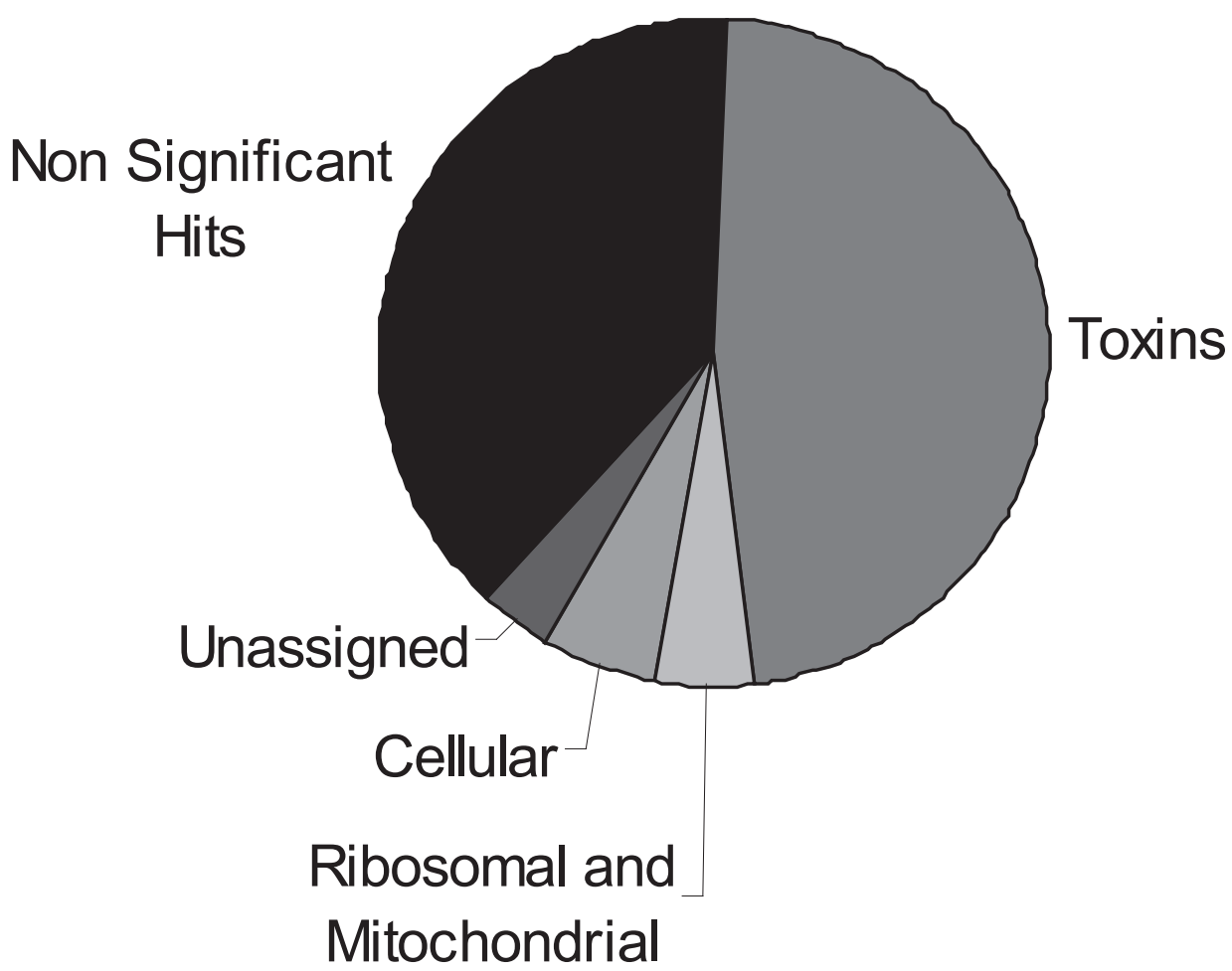

BLAST annotated composition of the *E. ocellatus* venom gland EST database. Relative composition of toxin, cellular, and ribosomal/mitochondrial transcripts are shown. Transcripts with no significant match to Uniprot and Trembl databases (non-significant hits) are also indicated. Transcripts whose potential function could be either intra or extracellular were classified as unassigned.
