# Supplementary material for: Bioinformatics and Multiepitope DNA Immunization to Design Rational Snake Antivenom
Source: PLoS Med. 2006 Jun 6;3(6):e184. doi: 10.1371/journal.pmed.0030184 (PMC1472699; doi:10.1371/journal.pmed.0030184)
Supplement: Supplementary file 2 [file pmed.0030184.sg002.doc]

Figure S1: Comparative sequence analysis of the full-length PI-PIII EoSVMP isoforms.

Propeptide Domain

Cysteine-rich Domain

Disintegrin Domain

Metalloproteinase Domain

Zn (MP-1)

I-Bi (MP 2 and 4)

I-Bii (MP-5)

MP-6

MP-3

PIII SVMPs lack Cys-195, postulated to predispose them to proteolytic processing (Fox and Serrano, 2005). EOC00001 contains an additional cysteine (Cys-367) shared with dimeric PIII SVMPs (Fox and Serrano, 2005). The additional unpaired Cys-582 in EOC00022 and 24 may facilitate the addition of a C-type lectin domain of PIV SVMP isoforms (Fox and Serrano, 2005; Hi*te et a*l., 1994).
