# Supplementary material for: Correction: Bioinformatics and Multiepitope DNA Immunization to Design Rational Snake Antivenom
Source: PLoS Med. 2008 Oct 28;5(10):e209. doi: 10.1371/journal.pmed.0050209 (PMC2573912; doi:10.1371/journal.pmed.0050209)
Supplement: Figure S1 — (48 KB PDF). [file pmed.0050209.sg001.pdf]

Figure S1: Comparative sequence analysis of the full-length PI-PIII EoSVMP isoforms.

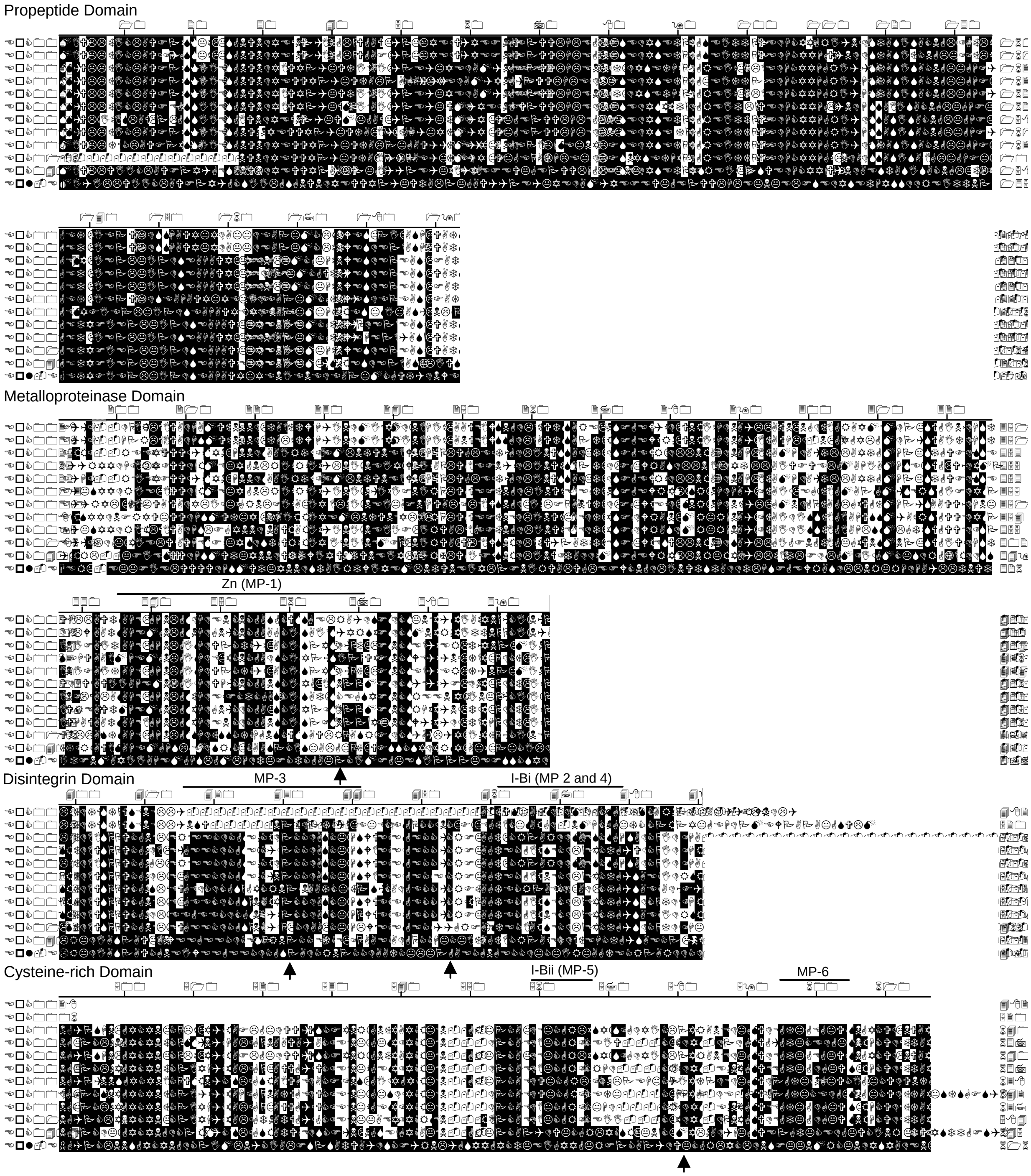


PIII SVMPs lack Cys-195, postulated to predispose them to proteolytic processing (Fox and Serrano, 2005). EOC00001 contains an additional cysteine (Cys-367) shared with dimeric PIII SVMPs (Fox and Serrano, 2005). The additional unpaired Cys-582 in EOC00022 and 24 may facilitate the addition of a C-type lectin domain of PIV SVMP isoforms (Fox and Serrano, 2005; Hite *et al.*, 1994).
