# Supplementary material for: Correction: Bioinformatics and Multiepitope DNA Immunization to Design Rational Snake Antivenom
Source: PLoS Med. 2008 Oct 28;5(10):e209. doi: 10.1371/journal.pmed.0050209 (PMC2573912; doi:10.1371/journal.pmed.0050209)
Supplement: Figure S2 — Found at doi:10.1371/journal.pmed.0050209.sg002 (32 KB PDF). [file pmed.0050209.sg002.pdf]

Figure S2: Composition of the *E.ocellatus* venom gland transcriptome

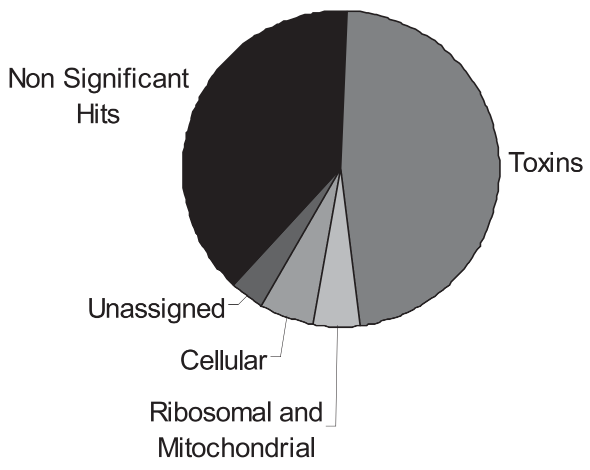

BLAST annotated composition of the *E. ocellatus* venom gland EST database. Relative composition of toxin, cellular, and ribosomal/mitochondrial transcripts are shown. Transcripts with no significant match to Uniprot and Trembl databases (non-significant hits) are also indicated. Transcripts whose potential function could be either intra or extracellular were classified as unassigned.
